# Effectiveness of an integrated multidisciplinary geriatric rehabilitation programme for older persons with stroke: a multicentre randomised controlled trial

**DOI:** 10.1186/s12877-021-02082-4

**Published:** 2021-02-23

**Authors:** Tom P. M. M. Vluggen, Jolanda C. M. van Haastregt, Frans E. Tan, Jeanine A. Verbunt, Caroline M. van Heugten, Jos M. G. A. Schols

**Affiliations:** 1 Department of Health Services Research, Maastricht University, Box 616, 6200 Maastricht, MD The Netherlands; 2 Care and Public Health Research Institute, Maastricht University, Maastricht, The Netherlands; 3 Department of methodology and statistics, Maastricht University, Maastricht, The Netherlands; 4 Adelante, Centre of Expertise in Rehabilitation and Audiology, Hoensbroek, The Netherlands; 5 Department of Rehabilitation Medicine, Maastricht University, Maastricht, The Netherlands; 6 Department of Neuropsychology and Psychopharmacology, Maastricht University, Maastricht, The Netherlands; 7 School for Mental Health and Neuroscience, Maastricht University, Maastricht, The Netherlands

**Keywords:** Stroke, Geriatric rehabilitation, Elderly persons, Randomised controlled trial, Aftercare

## Abstract

**Background:**

Almost half of the stroke patients admitted to geriatric rehabilitation has persisting problems after discharge. Currently, there is no evidence based geriatric rehabilitation programme available for older stroke patients, combining inpatient rehabilitation with adequate ambulatory aftercare in the community. Therefore, we developed an integrated multidisciplinary rehabilitation programme that includes aftercare for older persons with stroke. We evaluated the effectiveness of this newly developed rehabilitation programme in comparison to usual care.

**Methods:**

A multicentre randomised controlled trial was conducted in eight geriatric rehabilitation stroke units and their collaborating partners in primary care. The study population involved stroke patients and their informal caregivers who were aged 65 or over, living in the community before admission to geriatric rehabilitation, and expected to be able to return home after discharge. The programme consisted of three modules: inpatient neurorehabilitation, home-based self-management training, and stroke education. For patients, daily activity (FAI) was assessed as primary outcome and functional dependence (Katz-15), perceived quality of life (SSQoL) and social participation (IPA) as secondary outcomes. Additionally, among informal caregivers perceived care burden (self-rated burden VAS), objective care burden (Erasmus iBMG), and quality of life (CarerQol), were assessed as secondary outcomes.

**Results:**

In total 190 patients and 172 informal caregivers were included. Mean age of the patients in the intervention group was 78.9 years (SD = 7.0) and in the usual care group 79.0 years (SD = 6.5). Significant favourable effects for the programme were observed for the subscale autonomy outdoors of the IPA (− 2.15, *P* = .047, and for the informal caregivers perceived care burden (1.23, *P* = .048. For the primary outcome daily activity and the other secondary outcomes, no significant effects were observed.

**Conclusion:**

The integrated multidisciplinary programme had no effect on daily activity of older stroke patients. However, patients participating in the programme had a higher level of perceived autonomy of outdoor activities and their informal caregivers perceived a lower care burden. The programme might be promising in providing adequate (after) care, although adaptation of the programme is recommended to increase its feasibility and improve its effects.

**Trial registration:**

Current Controlled Trials ISRCTN62286281. Registered 19-3-2010.

**Supplementary Information:**

The online version contains supplementary material available at 10.1186/s12877-021-02082-4.

## Background

Stroke is one of the leading causes of death and a major cause of disability worldwide. Because of the aging population stroke is highly prevalent and can have a major impact on daily functioning and quality of life [1, 2].

In the Netherlands, each year about 40% of the older persons who suffer from acute stroke are admitted to an intermediate care facility for geriatric rehabilitation after a period of hospitalisation [3–5]. About half of the older stroke patients who are discharged home after geriatric rehabilitation still experience serious impairments in daily functioning and social participation, caused by severe cognitive and functional incapacities [6]. In patients who are socially inactive and are lacking appropriate coping skills, these impairments can lead to a substantial decrease in quality of life and depression [7]. Most older persons who are admitted to geriatric rehabilitation have multimorbidity that can interfere with rehabilitation and therefore may influence outcomes negatively. Besides a negative impact on patients, stroke and multimorbidity may also increase the burden of care perceived by informal caregivers which may also result in a decrease in their quality of life [7, 8]. Eventually, when the burden for the informal caregiver becomes too high, this may result in permanent admission of the patient to a long-term care facility.

In the Netherlands, stroke care for older patients is organised in stroke services aiming to realise more integrated care. This trend has led to a reduction in mortality, a decrease in admissions to long-term institutional care, more satisfaction among patients and caregivers, and more cost-effectiveness [9]. Although stroke care has achieved these quality improvements, sufficient aftercare after inpatient geriatric rehabilitation is often lacking in usual care, or when available, is too fragmented which makes it difficult to support patients and their informal caregivers in dealing with stroke related problems at home after discharge from rehabilitation.

Therefore, it seems important that older stroke patients and their caregivers, receive a rehabilitation treatment that includes tailor-made aftercare after discharge from geriatric rehabilitation to facilitate the transition to the home situation and to support patients and their caregivers in coping with the patients’ residual impairments in daily life. Training older patients and their caregivers in effective coping skills to manage their impairments might contribute to living independently in the community and staying socially active as long as possible. In addition, adequate aftercare may prevent negative long-term consequences such as decrease in daily activity level, depression and postpone admission to a long-term care facility [10, 11]. Therefore, stroke rehabilitation should include structural follow-up treatment in the patients’ home environment to improve functional independence of patients, to train patients in coping strategies to increase the adaptation skills to manage the remaining physical, cognitive and/or psychosocial impairments and improve quality of life, and to provide support for the informal caregiver to decrease the burden of care [12, 13].

Currently, there is no effective and well-organised aftercare programme available for older stroke patients admitted to geriatric rehabilitation [14]. Therefore, we developed an integrated multidisciplinary geriatric rehabilitation programme that includes aftercare for older persons with stroke. It aims to facilitate early discharge if possible, to train patients and informal caregivers to cope with the residual impairments by enhancing self-management, to optimise the level of participation after rehabilitation, and to provide support at home after discharge from rehabilitation.

The aim of this study was to evaluate the effects of this integrated programme as compared with usual care on the primary outcome daily activity level, and on the secondary outcomes functional independence, perceived quality of life and social participation of patients, and perceived care burden, objective care burden, and quality of life of their informal caregivers.

## Methods

### Study design

The design of this study was a two-arm multicenter randomised controlled trial with patients allocated to either the integrated programme or usual care. The study was conducted in eight geriatric rehabilitation units for patients with stroke. More specific information about the methodology of the study can be obtained from protocol article published earlier [15]. This study adheres to CONSORT guidelines for Randomized Controlled Trials. The protocol of this study was registered with the International Standard Randomised Controlled Trial Register Number (ISRCTN62286281), and The Dutch Trial Register (NTR2412).

### Study population

The study population involved stroke patients and their informal caregivers who were admitted to one of the eight participating geriatric rehabilitation stroke units after hospital discharge. The study population was restricted to patients aged 65 or over, living in the community before admission to geriatric rehabilitation, and expected to be able to return home after discharge. Inclusion started directly after admission to the geriatric rehabilitation unit. At admission the rehabilitation team under the responsibility of an elderly care physician, conducted a comprehensive geriatric assessment based on the Dutch Stroke guidelines to determine if the patient was expected to return home after discharge. The assessment includes measurements such as: age, sex, socio-economic status, risk factors, co-morbidity, stroke location and stroke severity measured by the National Institute of Health Stroke Scale, Barthel Index, Frenchay Activity Index, Modified Rankin Scale, Stroke Adapted Sickness Impact Profile 30, Mini Mental State Examination, Apraxia Test, Star Cancellation Test, Hospital Anxiety and Depression Scale and Caregiver Strain Index. Based on this assessment (in combination with the other eligibility criteria), patients were included in the study. Patients who did not give informed consent for participation, or were medically unstable and thereby not able to start rehabilitation, were excluded. In addition, the primary informal caregiver of each participating patient was invited to participate in the study. A person is considered to be the primary informal caregiver in case the patient indicates him/her as the person mostly involved in informal care activities for this patient. The multidisciplinary teams of the participating geriatric rehabilitation units received a 3 hour training which included the important key elements of the intervention protocol. During the study, the participating multidisciplinary teams were responsible for checking which admitted patients fulfilled the inclusion criteria of the study. To calculate the sample size, data from earlier research was used. Based on the Frenchay Activity Index score as primary outcome variable [16], the assumed clinically relevant difference in activity level of two stroke populations had to be at least 3.5. Based on a power of 0.8 and an alpha of 0.05, the study would need a sample size of 102 patients in each group. With an expected drop-out during follow-up estimated at approximately 25%, each group should include 128 participants. In total 256 participants were needed for the study.

### Randomisation

After inclusion, all patients and their informal caregivers of each participating nursing stroke unit were randomised on patient level by an independent research assistant. The randomisation procedure was conducted by a computerised block randomization schedule using IBM SPSS software version 19.0 (10 patients per block) to allocate the included patients to the intervention or usual care group. Patients allocated to the intervention group received the integrated programme and patients allocated to the usual care group received care as usual. Data were collected by research assistants who were blinded for treatment allocation. Because of study characteristics, blinding of patients, informal caregivers and care professionals involved was not possible.

### Integrated multidisciplinary geriatric rehabilitation programme

#### Organisation of the integrated programme

The integrated programme consisted of three care modules; 1) inpatient neurorehabilitation treatment; 2) home-based self-management training for patient and informal caregiver; and 3) stroke education for patient and informal caregiver. Table 1 presents both the integrated multidisciplinary geriatric programme and usual care.

**Table 1:** Integrated multidisciplinary geriatric rehabilitation programme and usual care

	Integrated programme	Usual care
**Care content**		
Multidisciplinary stroke team	+	+
Care based on Dutch stroke guidelines	+	+
Tailored approach with Goal Attainment Scaling	+	–
Self-management	+	–
Stroke education	+	–
Home therapy during nursing home admission	+	–
Multidisciplinary outpatient rehabilitation	+	–
Home visits of stroke care coordinator	+	–
**Care organisation**		
Stroke care coordinator	+	–
Multidisciplinary team meetings in nursing home	+	+
Multidisciplinary team meetings after discharge	+	–
Electronic patient record	+	–

The treatment progress was evaluated in monthly multidisciplinary team meetings for every individual patient. All communication and information by the care professionals about the patient and informal caregiver was conducted by using a shared electronic patient record, which was specifically developed for this study. To optimise care by facilitating faster discharge and to give support after discharge a stroke care coordinator was introduced in all participating rehabilitation teams. The total programme duration, including all three modules, varied between 2 to 6 months, depending on the care needs of the patient. All care professionals of the participating stroke teams were trained in conducting the programme according to protocol [15].

#### The stroke care coordinator

When the patient was admitted to the geriatric rehabilitation unit, the stroke care coordinator was introduced. The stroke care coordinator facilitated the transition of nursing home rehabilitation care services to community care by supporting the collaboration between the multidisciplinary stroke team of the nursing home and the community health services, namely community nurses, paramedical professionals and the general practitioner. After discharge, the coordinator conducted home visits, supports the general practitioner by organising multidisciplinary stroke team meetings and guided the patient and informal care giver in learning to apply self-management principles.

At the start of geriatric rehabilitation, the coordinator had an introduction meeting with both the patient and informal caregiver. In this meeting, the coordinator provided general information about the rehabilitation programme. Furthermore, during the rehabilitation process the coordinator facilitated the transition of the patient from inpatient geriatric rehabilitation care to home-based care by supporting the collaboration between the multidisciplinary stroke team of the geriatric rehabilitation unit, community health services and general practitioner. After discharge, the coordinator conducted at least two home visits, organised multidisciplinary stroke team meetings in the community and supported the patient and informal caregiver in practicing self-management skills at home.

#### Module 1: inpatient neurorehabilitation treatment for patients

The first module focused on (re) learning the abilities needed for individual patients to function as independently as possible in their own home environment. At the start of this module, an individual treatment plan was made together with the patient including the development of rehabilitation goals facilitating the transition from in-patient to home-based rehabilitation care and to guide further rehabilitation at the patient’s home.

To make rehabilitation goals more measurable during inpatient rehabilitation, the principles of the Goal Attainment Scaling (GAS) method were used. GAS is a methodology, which is shown to be appropriate for developing rehabilitation goals among older persons [16]. To facilitate transition to the home environment, during the stay at the geriatric rehabilitation unit, an occupational or physical therapist, depending on the rehabilitation goals, trained with the patients at least twice in their own home environment. These training sessions were done to optimise recovery, to train specific functional skills at home to increase independence, and to check if any home adjustments were needed before discharge. The training programme within this module was conducted by a multidisciplinary stroke team consisting of professionals working at the geriatric rehabilitation unit of the nursing home. The stroke rehabilitation team included an elderly care physician, a physical therapist, an occupational therapist, a speech therapist, a (neuro) psychologist and a stroke coordinator.

#### Module 2: home-based self-management training for patient and informal caregiver

The second module started directly after discharge to the home environment. Treatment focused on learning to cope with residual cognitive and functional impairments as a result of stroke. The stroke care coordinator trained patients and caregivers to improve their coping strategies and empowerment techniques. This training which included formulating rehabilitation goals for the patients and making action plans, were based on the basic principles of self-management and aimed to increase problem-solving skills and participation [13, 17]. If necessary, patients could still receive ambulatory follow-up rehabilitation treatment by a physical or occupational therapist, with the intention that at least half of the treatment sessions should take place in the patient’s home. If home treatment was not possible, the patient could receive this treatment in a day care facility or private therapy practice. The training in this module was also provided by the professionals of the regional multidisciplinary team consisting of professionals of the geriatric rehabilitation unit and community health care.

#### Module 3: stroke education for patient and informal caregiver

The third module was a short stroke education course for patients and their involved informal caregivers. The course consisted of four education sessions of 2 hours each with the focus on respectively the psychological and emotional consequences of stroke, perceived problems during independent living and participation in societal activities, and on the role of the informal caregiver. The course was provided by a (neuro) psychologist, two volunteers of the Dutch Stroke Patient Association and Informal Caregivers Association, and a social worker. The stroke coordinator invited the patients and informal caregivers to participate in the course. In two of the four meetings patients and informal caregivers were divided in two separate groups, to provide them the opportunity to express their problems and concerns more freely and share experiences with other patients/caregivers.

#### Usual care

In the Netherlands, usual care for older people with a stroke that need inpatient-rehabilitation consists of multidisciplinary neurorehabilitation on a geriatric rehabilitation unit. After discharge, there is in general no coordinated multidisciplinary aftercare for patient and informal caregiver. Most care programs vary in content and are in general more focused on the recovery of the patient and limited on the needs of the informal caregiver. After discharge, the follow-up care is usually provided by monodisciplinary community services, with no multidisciplinary approach. In general, there is no additional involvement anymore of the stroke rehabilitation team of the geriatric rehabilitation unit.

### Measurements

#### Background characteristics

The following background characteristics were measured in both patients and informal caregivers: age, sex, level of education, marital status, living situation, and relationship between patient and informal caregiver. In addition, cognitive functioning of patients was measured at baseline by means of the Mini Mental State Examination (consisting of 11-items, range 0–30 with higher scores indicating better functioning) [18–20].

#### Primary outcome measure

An overview of the primary and secondary outcome measurements per time point is presented in Table 2. Primary outcome measure was *daily activity* of patients measured by means of the Frenchay Activity Index (FAI) a 15-items activity scale (range 15–60 with higher scores indicating better functioning) [21]. The outcome of the FAI at baseline (i.e. at admission to the geriatric rehabilitation unit) was based on the activity level of patients 3 months before stroke occurred, as estimated by the patient. Follow-up measurements of the FAI were conducted after 6 and 12 months of follow-up.

**Table 2:** Overview of all outcome measures per time point

Subject	Outcome measures	Measurement scale	Number of items	Time point		
				T0	T1	T2
***Patient***	***Primary outcome measure***					
	Activity level after stroke	Frenchay Activity Index	15	FI	FI	FI
	***Secondary outcome measures***					
	Level of functioning	Katz-15	15	FI	FI	FI
	Stroke specific quality of life	Stroke Specific Quality of Life Questionnaire	49	FI	FI	FI
	Social participation	Impact on Participation and Autonomy (subscales autonomy outdoors and social life and relationships)	12	FI	FI	FI
***Informal caregiver***	***Secondary outcome measures***					
	Perceived care burden	Self-Rated Burden VAS	10	SQ	SQ	SQ
	Carer Quality of life	Carer Qol	7	SQ	SQ	SQ
	Objective care load	Erasmus iBMG	4	SQ	SQ	SQ
	***Background characteristics***					
	Background characteristics patient	-	10/5/5	FI	FI	FI
	Background characteristics informal Caregiver	-	8/7/7	SQ	SQ	SQ
	Cognitive functioning patient	Mini Mental State Examination	11	FI	–	–

T0 = at baseline, T1 = after 6 months, T2 = after 12 months, FI = face-to-face interview, SQ = self-report questionnaire

#### Secondary outcome measures

Secondary outcome measures for patients were *functional dependence* measured by the Katz-15 (consisting of 15 items (range 0–15 with lower scores indicating a higher level of independence) [22], *perceived quality of life* measured by the Stroke Specific Quality of Life scale (SSQoL) a 49-items quality of life scale which contains two subscales “Physical functioning” consisting of 27 items (range 27–135) and “Psychosocial functioning” consisting of 22 items (range 22–110). For both subscales of the SS-QOL, a lower score indicates a lower perceived quality of life [23]. The outcome measure *social participation* was measured by two subscales “Autonomy outdoors” consisting of 5 items (range 0–20) and “Social relations” consisting of 7 items (range 0–28)) of the Impact on Participation and Autonomy (IPA) [24]. For both IPA subscales a lower score indicates a better participation level. The other subscales of the IPA were excluded because of the overlap with items of the FAI, Katz-15, and SSQoL. The Katz-15, IPA, and SSQol were measured at admission to the geriatric rehabilitation unit, and after 6 and 12 months of follow-up.

Secondary outcome measures in informal caregivers involved the perceived care burden measured by means of the Self-Rated Burden VAS scale (10 points likert scale with lower scores indicating less care burden) and the Carer Quality of Life scale (7 items with lower scores indicating less care burden) [25], *objective care burden* measured by means of the Erasmus iBMG (4-items care burden scale; item 1 “time spent on helping patient with ADL-activities”, item 2 “time spent on helping patient with personal care”, item 3 “time spent on helping patient with moving outside”, item 4 “time spent by other informal caregivers or volunteers on helping patient”) [26]. The total amount of time spent on the four items indicates the dependence of help by the informal caregiver. All secondary outcome measures were measured at admission to the geriatric rehabilitation unit, and after 6 and 12 months of follow-up.

### Data collection

Data for the effect evaluation was collected by face-to-face interviews among patients and self-reported questionnaires among informal caregivers (see Table 2). Research assistants conducted the interviews in the geriatric rehabilitation unit and at the patient’s home and provided the self-administered questionnaires to caregivers at baseline, after 6 months and after 12 months. All data was gathered between 2010 and 2015.

### Statistical analyses

Background characteristics of the patients and informal caregivers were checked for meaningful imbalance, analysed and described by using descriptive statistics. Analyses of the difference between primary and secondary outcomes for intervention group and usual care group were performed according to the intention-to-treat principle (with possible covariates taken into account in case of observed imbalance in baseline characteristics),including all valid data of all available participants, regardless of whether they received the (complete) programme. A two-level linear regression analysis was performed to calculate differences between the intervention and usual care group with regard to primary and secondary outcome measures. In the analyses level one was the repeated measures and level two was the patients. In all analyses, *P* = < .05 was considered statistically significant. All statistical analyses were conducted using IBM SPSS software version 25 for Windows by a researcher who was blinded for treatment allocation.

### Ethics

The medical ethics committee of Maastricht University Medical Centre (MUMC+), the Netherlands, approved this study.

## Results

### Background characteristics

**Fig. 1:**
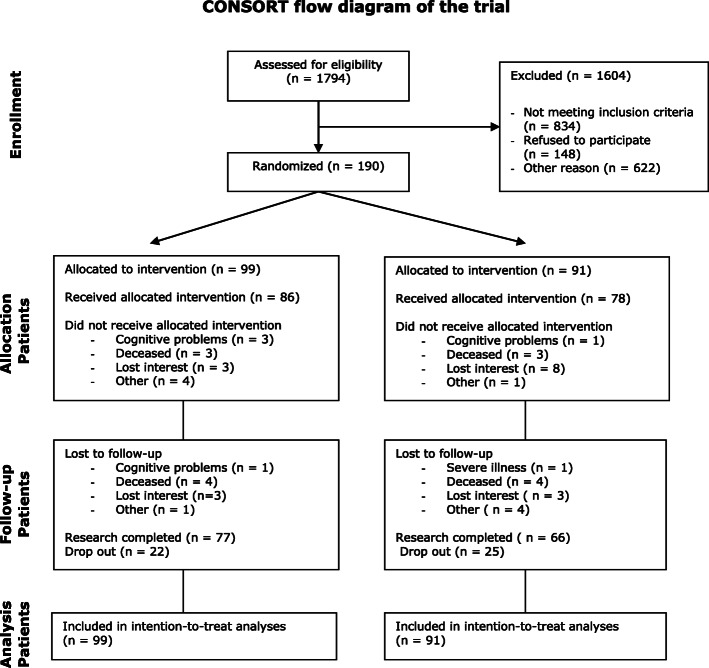
flowchart of patients through the study

Table 3 shows the background characteristics of patients and informal caregivers. In total 190 patients (mean age: 78.9 years) and 172 informal caregivers (mean age: 60.8 years) were included. Of these 190 patients 99 patients were randomised to the intervention group and 91 patients to the usual care group. Figure 1 shows the flowchart of the patient sample.

**Table 3:** Baseline characteristics of patients and informal caregivers

Baseline characteristics	Scores (N)	
***Patients (N = 190)***	*Intervention group (N = 99)*	*Usual care group (N = 91)*
***Background characteristics***		
Mean age (SD)	78.9 (7.0)	79.0 (6.5)
Female sex N (%)	69 (69.7)	46 (51.1)
Mean cognitive status (MMSE) (SD)	21.9 (5.2)	22.0 (4.1)
Maried with a partner N (%)	39 (40)	43 (47)
Living situation		
• Independent alone N (%)	53 (54.0)	43 (47.3)
• Independent with others N (%)	45 (45.5)	47 (51.6)
***Outcome measurement at baseline***	**Observed mean (SD)**	
*Primary outcome*		
Frenchay Activity Index (FAI)	40.2 (8.8)	38.8 (7.3)
*Secondary outcome*		
Impact on Participation and Activity (IPA)		
• Autonomy outdoors	15.4 (4.4)	14.8 (4.3)
• Relationship	15.3 (3.9)	15.8 (3.1)
Katz-15	6.0 (4.0)	6.5 (3.3)
Stroke Specific Quality of Life (SSQoL)		
• Subscale physical functioning	99.6 (20.8)	97.1 (21.3)
• Subscale psychosocial functioning	77.2 (16.8)	76.2 (16.9)
***Informal caregivers (N = 172)***	*Intervention group (N = 90)*	*Usual care group (N = 82)*
***Background characteristics***		
Mean age (SD)	61.0 (13.5)	60.5 (13.5)
Female sex N (%)	53 (64.6)	58 (70.7)
Relationship with patient		
*Husband, wife, life partner N (%)*	28 (31.1)	32 (39.0)
*Sister, brother, brother in law, sister in law N (%)*	3 (3.3)	5 (6.1)
*Daughter (in law), son (in law) N (%)*	56 (62.2)	40 (48.8)
*Other N (%)*	3 (3.3)	5 (6.1)
***Outcome measurement at baseline***	**Observed mean (SD)**	
Carer Quality of Life	85.9 (12.7)	82.5 (14.7)
Erasmus iBMG (time spended on helping patient)		
Item 1: helping patient with ADL-activities	0.5 (0.4)	0.5 (0.4)
Item 2: helping patient with personal care	0.8 (0.3)	0.8 (0.4)
Item 3: helping patient with moving outside	0.5 (0.4)	0.5 (0.4)
Item 4: help by other informal caregivers or volunteers	0.7 (0.4)	0.7 (0.4)
Self-Rated Burden VAS	4.0 (2.4)	4.3 (2.4)

Of the 172 informal caregivers 90 were randomised to the intervention group and 82 to the usual care. In both groups most informal caregivers were female. More than half of the patients lived alone, and the most common relationship between informal caregiver and the patient was a parent-child (in law) relationship. The analyses showed only an imbalance in sex (this outcome measure was included as a covariate), but further no meaningful imbalance between all other outcome measures of the intervention and usual care group at baseline (see Additional file 1: Baseline characteristics of patients checked on statistical differences at baseline).

### Effects of the integrated programme

#### Patients

Results of the two-level multilevel analysis on patient level are presented in Table 4. The results show that the intervention had no effect on the primary outcome daily activity as measured with the FAI (− 1.69, *p* = .368).

**Table 4:** Effects on primary and secondary outcomes in patients

***Variable***	***6 months follow-up observed mean (SD)***		***Group effect***	***P-value***	***95% CI***	***ICC***
*Primary outcome*						
	Intervention group	Usual care group				
Frenchay Activity Index (FAI)	29.7 (8.9)	29.1 (9.1)	−1.69	.368	−5.39 – 2.00	.74
*Secondary outcomes*						
Impact on Participation and Activity (IPA)						
• Autonomy outdoors	14.2 (4.1)	14.6 (4.2)	−2.15	.047	−4.27 – - .03	.55
• Social relations	15.2 (3.4)	16.0 (4.1)	.60	.560	−1.43 – 2.63	.41
Katz-15	5.9 (3.5)	6.0 (4.0)	−.69	.372	−2.22 - .83	.69
Stroke Specific Quality of Life (SSQoL)						
• Subscale physical functioning	102.2 (19.9)	99.2 (22.5)	3.08	.476	−5.41 – 11.56	.71
• Subscale psychosocial functioning	81.3 (17.1)	78.7 (18.0)	8.45	.054	−.14–17.03	.64

The analyses did show a significant favourable effect for the intervention on the subscale “Autonomy outdoors” of the IPA scale (− 2.15, *p* = .047). All other secondary outcome measures i.e. subscale “Social relations” of the IPA scale (.60, *p* = .560), Katz-15 questionnaire (−.69, *p* = .372), subscale “Physical functioning” of the SSQoL scale (3.08, *p* = .476), and the subscale “Psychosocial functioning” of the SSQoL scale (8.45, *p* = .054) showed no significant effects..

### Informal caregivers

Table 5 presents the results of the effect of the intervention on the informal caregiver. The results show that the intervention had a significant favourable effect on the Self-Rated Burden vas scale (1.23, *p* = .048), but no effects on the other outcome measures Carer Quality of Life questionnaire (3.54, *p* = .323), and Erasmus iBMG; item 1) helping patient with ADL-activities (.09, *p* = .447), item 2) helping patient with personal care (.09, *p* = .380), item 3) helping patient with moving outside (−.03, *p* = .784), item 4) help by other informal caregivers of volunteers (.03, *p* = .767).

**Table 5:** Effects on the outcomes in informal caregivers

***Variable***	***6 months follow-up observed mean (SD)***		***Group effect***	***P-value***	***95% CI***	***ICC***
	Intervention group	Usual care group				
Carer Quality of Life	85.3 (11.6)	82.9 (13.9)	3.54	.323	−3.50 – 10.58	.41
Erasmus iBMG (time spended on helping patient)						
Item 1: helping patient with ADL-activities	0.4 (0.4)	0.4 (0.4)	.09	.447	−.15 – .34	.49
Item 2: helping patient with personal care	0.8 (0.4)	0.8 (0.4)	.09	.380	−.12 – .30	.44
Item 3: helping patient with moving outside	0.4 (0.3)	0.5 (0.4)	−.03	.784	−.25 – .19	.38
Item 4: help by other informal caregivers or volunteers	0.6 (0.4)	0.6 (0.4)	.03	.767	−.18 – .24	.55
Self-Rated Burden VAS	4.3 (2.3)	4.0 (2.0)	1.23	.048	−.02 – 2.48	.43

## Discussion

The results of this study show that the integrated multidisciplinary geriatric rehabilitation programme for older patients with stroke had no significant effect on the primary outcome daily activity as compared to usual care. With regard to the secondary outcomes, the programme showed favourable effects on the patients’ outdoor autonomy and the perceived care burden of their informal caregivers. For the other secondary outcomes, no significant intervention effects were observed.

The lack of effect of the programme on daily activity and most secondary outcome measures might be explained by several reasons. First, the process evaluation which was performed alongside the trial, revealed that part of patients and informal caregivers did not receive all key elements of the programme [27]. Although almost all patients formulated rehabilitation goals, the GAS method was only used among two thirds of the patients. In addition, the percentage of therapy sessions performed in the patients’ home environment was lower than planned, and only about a quarter of the patients and informal caregivers attended the education sessions. Furthermore, the self-management training was considered by the care professionals as rather complex and difficult to apply for frail older persons, because it was complicated for the patients to develop and carry out action plans by themselves [27]. As it is widely recognised that in complex interventions often not all aspects of the intervention are completely performed according to protocol and that adaptation to local circumstances may be necessary [28], it is important to improve the feasibility of the integrated programme by tailoring the goal attainment scaling, self-management training and education sessions more optimally to the population of frail older stroke patients [27]. In addition the training of care professionals in conducting the programme could be improved. However, despite this, the majority of patients, informal caregivers and care professionals indicated the beneficial aspects of the programme [27].

Second, a review of Fens and colleagues [29] performed in 2013 evaluating the effectiveness of multidisciplinary interventions for stroke patients living in the community after being discharged home after hospitalization or inpatient rehabilitation, showed that none of the 11 studies that assessed daily activities reported a favourable effect of the intervention on this outcome. Although these multidisciplinary interventions included different combinations of elements, it clearly shows that improving daily activity among community living stroke patients is very complex, which is also confirmed by the results of our trial.

Based on our results, the increased level of autonomy outdoors of the patients receiving the programme, seems to indicate that despite the lack of increase in the actual frequency of daily activity as measured by the FAI, the level of (outdoor) activities is more in accordance with the needs and wishes of the patients. An explanation for this finding could be that the self-management component of the programme may have improved the coping skills of patients and their informal caregivers, and helped them to have more realistic expectations about the patients’ outdoor activities. The increase in autonomy related to outdoor activities, is an important finding, as De Graaf and colleagues emphasised the need to pay more attention to the social participation of stroke survivors aged over 70 years, since more restrictions in participation were perceived in comparison to younger stroke survivors 1 year after stroke [30]. Furthermore, increased attention for participation may also contribute to preventing depressive symptoms after stroke [31].

With regard to the informal caregivers, the integrated programme resulted in a significant reduction in the perceived care burden of the informal caregiver. This may indicate that elements of the integrated programme, such as consultation with the stroke coordinator and stroke education, may support informal caregivers in accomplishing their supporting role. This is in accordance with the results of a review of Visser-Meily and colleagues [32] who concluded that counselling programs which focus on the problems of the informal caregiver, instead of (only) on the problems of the patients, appear to have the most favourable outcomes. In our programme, the problems and experiences of the informal caregiver were explicitly addressed in different modules.

This study is one of few studies that focusses on improving stroke rehabilitation and aftercare for frail older stroke patients and their informal caregivers. However, this study has several limitations. First, we did not reach our inclusion goal of 256 patients, although we took all possible and necessary actions (i.e. extending inclusion period, extending the number of nursing homes) to increase the number of patients. This may have underpowered our multilevel analyses. However, the estimated difference between intervention and usual care group on our primary outcome daily activity (i.e. 1.69) is below the minimal effect that is still considered clinically relevant (i.e.3.5). Therefore, it is unlikely that including the intended number of patients would have resulted in a statistically significant effect on our primary outcome. However, for the psychosocial functioning subscale of the Stroke Specific Quality of life scale (*p* = .054) accounts that a higher power may have resulted in a statistical significant favourable effect for patients in the intervention group on this subscale.

Second, because we randomised on patient level and not on nursing home level, care professionals treated both people in the intervention group and usual care group. Therefore, it is possible that treatment for persons in the usual care group was contaminated with elements of the programme which may have led to an underestimation of the effects of the programme. Although a number of elements of the programme were exclusively available for persons in the intervention group (such as the meetings with the stroke care coordinator, the multidisciplinary outpatient rehabilitation, and the stroke education course), it is still possible that other elements of the intervention were also applied among persons in the usual care group. However, we have tried to reduce this risk of contamination by emphasizing during the training of the care professionals that the programme elements should exclusively be applied in the intervention group. In addition we repeatedly checked whether contamination has occurred during regular visits of the research team to the participating organisations. During these visits care professionals confirmed that the intervention was only applied to persons in the intervention group. Furthermore, after the intervention period, we checked during a group interview with a sample of the participating care professionals whether contamination had occurred, which was not the case according to the care professionals.

Third, patients, caregivers and care professionals could not be blinded for treatment allocation, which might have created some bias. However, in order to reduce the risk of any additional bias, the outcome measurements were performed by research assistants who were blinded for treatment allocation, and the same accounts for the statistical analyses.

Fourth, there could have been interference by possible language disturbances caused by stroke. Although we examined cognition by the MMSE we cannot rule out the fact that possible aphasic syndromes may have caused interference because we did not conduct a specific language assessment for stroke. Despite that, randomisation limited the chance that any possible language disturbance in our population influenced our results.

Fifth, the baseline measurement of the FAI was based on the activity level of patients 3 months before stroke occurred, as estimated by the patient. It is possible that this resulted in recall bias. However, it is likely that this accounts for patients in both the usual care and intervention group, which makes it unlikely that is has influenced our results.

## Conclusion

This study shows that an integrated multidisciplinary rehabilitation programme for frail older patients with stroke and their caregivers had no effect on the activity level of these patients. However, the intervention did show a significant favourable effect on autonomy regarding outdoor activities as perceived by the patients. Furthermore, we found also a significant favourable effect on the perceived care burden of informal caregivers. Based on these results, the programme might be considered promising in providing adequate aftercare. However, adaptation of the programme is recommended to increase its feasibility and to improve its favourable effects for patients and informal caregivers. More research is needed to increase knowledge and evidence of effective methods to increase daily activity level in (older) patients with stroke.

## Supplementary Information

**Additional file 1.**

## Data Availability

The datasets used and/or analysed during the current study are available from the corresponding author on reasonable request.
