# Supplementary material for: Effectiveness of an integrated multidisciplinary geriatric rehabilitation programme for older persons with stroke: a multicentre randomised controlled trial
Source: BMC Geriatr. 2021 Feb 23;21:134. doi: 10.1186/s12877-021-02082-4 (PMC7903755; doi:10.1186/s12877-021-02082-4)
Supplement: Supplementary file 1 — Additional file 1. [file 12877_2021_2082_MOESM1_ESM.docx]

| **Baseline characteristics** | **Scores (N)** | |  |
| --- | --- | --- | --- |
|  |  |  |  |
| ***Patients (N=190)*** | *Intervention group (N=99)* | *Usual care group (N=91)* | *P-value* |
|  |  |  |  |
| ***Background characteristics*** |  |  |  |
| Mean age (SD) | 78.9 (7.0) | 79.0 (6.5) | 0.92 |
| Female sex N (%) | 69 (69.7) | 46 (51.1) | 0.007 |
| Mean cognitive status (MMSE) (SD) | 21.9 (5.2) | 22.0 (4.1) | 0.90 |
| Maried with a partner N (%) | 39 (40) | 43 (47) | 0.19 |
| Living situation | | |  |
| - Independent alone N (%) | 53 (54.0) | 43 (47.3) | 0.69 |
| - Independent with others N (%) | 45 (45.5) | 47 (51.6) | 0.69 |
|  |  | |  |
| ***Outcome measurement at baseline*** | **Observed mean (SD)** | | |
| *Primary outcome* | | |  |
| Frenchay Activity Index (FAI) | 40.2 (8.8) | 38.8 (7.3) | 0.23 |
| *Secondary outcome* | | | |
| Impact on Participation and Activity (IPA) |  |  |  |
| - Autonomy outdoors | 15.4 (4.4) | 14.8 (4.3) | 0.34 |
| - Relationship | 15.3 (3.9) | 15.8 (3.1) | 0.26 |
| Katz-15 | 6.0 (4.0) | 6.5 (3.3) | 0.22 |
| Stroke Specific Quality of Life (SSQoL) |  |  |  |
| - Subscale physical functioning | 99.6 (20.8) | 97.1 (21.3) | 0.46 |
| - Subscale psychosocial functioning | 77.2 (16.8) | 76.2 (16.9) | 0.69 |

**Appendix: Baseline characteristics of patients checked on statistical differences at baseline**
